# Determinants of willingness to receive healthy lifestyle advice in the context of cancer screening

**DOI:** 10.1038/s41416-018-0160-4

**Published:** 2018-07-11

**Authors:** Claire Stevens, Charlotte Vrinten, Samuel G. Smith, Jo Waller, Rebecca J. Beeken

**Affiliations:** 1 Department of Behavioural Science and Health, University College London, London, WC1E 6BT UK; 2 Leeds Institute of Health Sciences, University of Leeds, Leeds, LS2 9NL UK

**Keywords:** Lifestyle modification, Population screening

## Abstract

**Background:**

Providing lifestyle advice at cancer screening may help reduce the cancer burden attributable to health-related behaviour. We examined determinants of willingness to receive advice about several behavioural cancer risk factors.

**Methods:**

A population-based sample of English adults eligible for cancer screening (*n* = 1221) completed items on willingness to receive lifestyle advice. Sociodemographic, psychological (risk perceptions, cancer risk factor awareness) and behavioural factors were used to predict interest in advice about diet, weight, physical activity, smoking and alcohol consumption.

**Results:**

Two thirds (62–67%) reported interest in advice about diet, weight, and physical activity; 17% were willing to receive advice about smoking, and 32% about alcohol consumption. Willingness to receive advice was higher in those not adhering to guidelines for weight, physical activity, smoking and alcohol consumption (all *p* < 0.01). Non-White ethnicity was associated with interest in advice about diet, physical activity and smoking (all *p* < 0.01). Willingness to receive advice about diet, weight, physical activity and alcohol consumption increased with greater recognition of cancer risk factors (all *p* < 0.01).

**Conclusions:**

Willingness to receive lifestyle advice at cancer screening was high, suggesting this context may provide an opportunity to support behaviour change. Increasing awareness of cancer risk factors may facilitate interest in lifestyle advice.

## Introduction

It is estimated that exposure to lifestyle and environmental risk factors, such as smoking, dietary factors and overweight account for 43% of cancers.^1^ The importance of behavioural risk factors for cancer is recognised in cancer strategy documents.^2,3^ However, a large proportion of English adults are at increased risk of developing cancer as they fail to meet current health behaviour recommendations.^4,5^

The cancer screening context could provide ‘teachable moments’ for the delivery of behaviour change advice and interventions.^6^ Delivering behaviour change advice within existing health care services is consistent with government policy to ‘Make Every Contact Count’.^7^ Lifestyle advice is not routinely offered alongside cancer screening, but this setting could provide an opportunity to reach a large number of people in both primary and secondary care.^8^. We have previously shown that three quarters of people intending to attend English breast, cervical and bowel screening programmes would be in favour of receiving lifestyle advice around the time of cancer screening, even if they received an abnormal result (CS, CV, SGS, JW & RJB; under review). Interventions delivered at the time of breast and bowel cancer screening have reported adequate uptake and positive health behaviour change.^9,10^ One concern about delivering lifestyle advice in the cancer screening context is the potential negative effect it may have on screening uptake; 6–9% reported advice would be a deterrent in our previous study (CS, CV, SGS, JW & RJB; under review). Understanding the sociodemographic, psychological and behavioural determinants of interest in healthy lifestyle advice at cancer screening may aid the development of effective interventions which minimise negative effects on screening uptake.

There is sociodemographic variation in the uptake of English cancer screening programmes.^11,12^ Uptake of Flexible Sigmoidoscopy is lower (33%) in the most deprived areas, compared with the least deprived areas (53%).^11^ In addition, ethnic minority groups are less likely to participate in cancer screening than white populations.^13–15^ Similar sociodemographic variation has been observed for other cancer related health behaviours including smoking and diet.^16,17^ Less is known about whether there is also variation in receptivity to different topics of lifestyle advice at cancer screening. Some research has found that interest in advice at breast screening was higher in women who were older, less educated and overweight.^18^ However, other research has found that receptivity to advice at cervical screening may be greater among more educated and non-White populations (CS, CV, SGS, JW & RJB; under review). One lifestyle intervention evaluated within a bowel screening setting reported high levels of recruitment and retention from deprived groups, and found no differences in outcomes between more and less deprived groups.^19^

Risk perceptions are commonplace in theories attempting to understand and change health behaviour.^20^ There is evidence to suggest changing perceptions of risk may affect intentions and behaviour.^21^ Risk perceptions are a key component of the Teachable Moment Heuristic.^22^ The model states that for behaviour change to occur following the teachable moment, perceived risk must increase. It has also been suggested that to capitalise on the teachable moment, people must be aware of the risk factors for cancer and how they relate to their own behaviour.^8^ A survey of British adults found variation in the recognition of different risk factors for cancer.^23^ Recognition was high for smoking (98%), but lower for other important risk factors such as overweight (62%) and fruit and vegetable consumption (48%). Awareness of the link between lifestyle and cancer could be a determinant of interest in lifestyle advice at cancer screening.^8^ Furthermore, receptivity to lifestyle advice may increase if people are given convincing evidence showing a link between lifestyle and cancer-related outcomes.^24^

This research aimed to gauge public interest in advice about five different aspects of lifestyle at cancer screening: diet, weight, physical activity, smoking, and alcohol consumption. In addition, this research aimed to identify sociodemographic, psychological (risk perception, cancer risk factor awareness) and behavioural predictors of interest in each of the lifestyle advice topics.

## Methods

### Design

Cross-sectional population-representative data were collected as part of the Attitudes, Behaviour and Cancer UK Survey (ABACUS). This survey aims to explore the determinants of early detection and prevention behaviours related to cancer. Data were collected as part of an omnibus survey, conducted by market research company Taylor Nelson Sofres (TNS) in April and May 2016 using home-based computer-assisted personal interviews.

### Participants

A nationally representative sample of 2048 English adults (aged 18–70) was recruited using stratified random location sampling, based on 2011 Census data and Postcode Address File data. Quotas were set for gender, working status and presence of children in the home. This research included a sub-sample of men and women currently eligible to participate in breast, bowel or cervical screening, and people approaching the age of eligibility. We included people approaching the age of eligibility for cancer screening for two reasons. Firstly, if lifestyle advice is routinely offered alongside cancer screening it is important to sample potential future attenders as well as people currently eligible to attend. Secondly, the narrow age range of patients invited to take part in flexible sigmoidoscopy and delays in the roll out of this screening programme would have limited our sample. Participants were included in the analysis if they intended to take part in at least one of the cancer screening programmes. Taking into account recent changes to cancer screening programmes, people classified as currently eligible for cancer screening were women aged 25–70, and men aged 60–70, and people classified as approaching eligibility were women aged 18–24 and men aged 45–59. Three cancer screening programmes operate in England. Women are invited to attend cervical screening between the ages of 25–64, and breast screening between the ages of 50–70. Men and women are invited to take part in Faecal Occult Blood testing (FOBT) between the ages of 60–74, and a one off Flexible Sigmoidoscopy (FS) aged 55. The upper age limit for FOBT eligibility has recently increased from 69 to 74, and FS was not available in all parts of England at the time of this survey. After excluding participants with a diagnosis of cancer (*n* = 121), people who did not meet age requirements (*n* = 471), and people who did not intend to attend a cancer screening programme in the future (*n* = 235), there was a final sample of 1221.

### Measures

#### Sociodemographic variables

Participants’ age, gender, ethnicity, and educational attainment (as a marker of socioeconomic status) were recorded. For analyses, ethnicity was categorised into White and non-White. Educational attainment was categorised into ‘degree level or above’ and ‘education below degree level’, based on the item ‘what is the highest level of educational qualification you have obtained’.

#### Cancer screening intention

Intention to participate in cancer screening was assessed separately for each of the four cancer screening programmes (breast, cervical, FOBT, FS; e.g. ‘Will you go for breast screening when, or next time you are invited?’). Four response options were offered (Yes, definitely; Yes, probably; No, probably not; No, definitely not), which were dichotomised into yes and no. A composite item was created which identified participants intending to take part in at least one of the screening programmes in the future.

#### Knowledge of cancer risk factors

Knowledge of cancer risk factors was assessed using an 11-item scale from the Cancer Research UK Cancer Awareness Measure (CRUK CAM^25^). Participants were presented with 11 risk factors for cancer including smoking, exposure to another person’s cigarette smoke, alcohol consumption, fruit and vegetable consumption, red and processed meat consumption, overweight, childhood sunburn, age over 70 years, having a close relative with cancer, infection with HPV, and physical inactivity. Five response options were provided, which were categorised into correct (agree / strongly agree) or incorrect (strongly disagree / disagree / not sure). For each participant, the number of risk factors that they correctly identified was combined giving each participant a score out of 11.

#### Comparative cancer risk

Comparative cancer risk perception was assessed using the item ‘How would you rate your chances of getting cancer, compared with other men / women your age?’, adapted from existing measures.^26,27^ Five response options were categorised into lower (much lower / a little lower), the same (about the same), and higher (a little higher / much higher).

#### Current health behaviours

Fruit and vegetable consumption was assessed using two items;^28^ *‘*Over the past month, how many portions of fruit / vegetables did you usually eat?’ Responses options were: less than 1 per week, 1 per week, 2–3 per week, 4–6 per week, 1 per day, 2 per day, 3 or more per day. Values ≥1 or more per day for each item were added together to create a composite measure of fruit and vegetable consumption. Participants consuming five or more portions of fruit and vegetables per day were classified as meeting guidelines. Body Mass Index (BMI; kg/m^2^) was calculated from self-reported height and weight. Implausible BMI data were excluded (BMI < 14 /> 50). BMI was dichotomised to ≥25 (overweight) vs <25 (not overweight). A single item was used to assess levels of physical activity ‘In the past week on how many days have you done a total of 30 minutes or more of physical activity, which was enough to raise your breathing rate?’.^29,30^ Participants taking part in 30 min of physical activity on five or more days per week were classified as meeting guidelines.^31^ Smoking status was assessed using a single item; ‘Do you smoke at all nowadays?’. Participants were categorised as smokers (Yes, I smoke daily; Yes, I smoke occasionally) or non-smokers (Not now, but I used to smoke daily; Not now, but I used to smoke occasionally; I have tried smoking in the past, but have never been a smoker; I have never smoked). Alcohol consumption items were adapted from the AUDIT-C questionnaire.^32,33^ ‘In a typical week, on how many days do you have a drink containing alcohol?’ and ‘How many units of alcohol do you drink on a typical day when you are drinking?’ Participants consuming 14 units or less per week were classified as meeting guidelines for alcohol consumption.

#### Willingness to receive different types of lifestyle advice at cancer screening

The following questions were asked of participants intending to take part in at least one screening programme in the future; ‘At cancer screening / If you were to attend cancer screening in the future, how interested would you be in any information or advice to… Help you have a healthy diet / Help you maintain a healthy weight / Help you increase your physical activity / Help you stop smoking / Help you reduce your alcohol consumption?’. Five response options were collapsed into ‘interested’ (a little interested / somewhat interested / very interested) and ‘not interested’ (not at all interested / not applicable).

### Analyses

Five adjusted logistic regression models explored sociodemographic, psychological and behavioural predictors of interest in each of the categories of lifestyle advice among people intending to participate in cancer screening in the future. The simultaneous entry method was used and each model included variables which have been associated with health behaviours and cancer screening participation: age, gender, ethnicity, educational attainment, screening eligibility, cancer risk factor recognition, comparative cancer risk, and current health behaviours. We applied survey weights calculated by the market research company to adjust for response bias (based on age, region, social grade, and working status). Sample characteristics are presented unweighted and weighted, univariate analyses are presented weighted, multivariate analyses are presented unweighted. Participants with a previous diagnosis of cancer and those who did not intend to take part in screening were excluded from analyses. Data were analysed using Stata SE 14. An alpha level of *p* < 0.010 was used to adjust for multiple testing.

## Results

### Sample characteristics

Of the 2048 adults included in the ABACUS, 1221 were included in this analysis (Table 1). The mean age of the sample was 46.9 (SD 15.1). Three quarters (73.8%, *n* = 901) were female, which reflects the inclusion criteria based on screening eligibility. The majority (87.9%, *n* = 1070) were White. One third of the sample (32.1%, *n* = 375) were educated to degree level or above. Three quarters of the sample (75.1%, *n* = 917) were currently eligible to participate in at least one cancer screening programme.

**Table 1:** Sample characteristics

	Weighted		Unweighted	
	**(***n* = 1221)		**(***n* = 1250)	
	M	SD	M	SD
*Age*				
	46.9	15.1	46.6	15.7
	*n*	%	*n*	%
*Gender*				
Male	320	23.2	941	24.7
Female	901	73.8	309	75.3
*Ethnicity*				
White	1070	87.9	1090	87.5
Non-White	148	12.1	156	12.5
*Education*				
Degree level or above	375	32.1	331	27.8
Qualifications below degree level	792	67.9	861	72.2
*Eligibility for screening*				
Currently eligible	917	75.1	978	78.2
Approaching eligibility	304	24.9	272	21.8
*Adherence to health behaviour guidelines*				
Fruit and vegetable consumption	452	37.2	436	35.1
BMI	499	48.8	500	48.3
Physical activity	369	30.4	375	30.2
Smoking	1028	84.6	1038	83.4
Alcohol consumption	1054	88.5	1088	89.3
*Comparative cancer risk*				
Higher	166	14.1	176	14.7
Same	711	60.5	726	60.7
Lower	298	25.4	295	24.6
	M	SD	M	SD
*Cancer risk factor recognition (out of 11)*				
	5.9	2.7	5.7	2.7

The majority of the sample did not smoke (84.6%, *n* = 1028), and consumed fewer than 14 alcoholic units per week (88.5%, *n* = 1054). Half of the sample had a BMI of 25 or lower (48.8%, *n* = 499). Around one third reported meeting guidelines for fruit and vegetable consumption (37.2%, *n* = 452) and physical activity (30.4%, *n* = 369). Most of the sample reported their risk of developing cancer to be the same as others of their age and sex (60.5%, *n* = 711). On average, people were able to recognise 5.9 (SD 2.7) of the 11 cancer risk factors. For unweighted sample characteristics, see Table 1.

### Interest in lifestyle advice at cancer screening

Two thirds of participants were interested in receiving advice about diet (67.3%, *n* = 799), weight (65.9%, *n* = 782) or physical activity (61.5%, *n* = 727) during future cancer screening appointments (Table 2). Around one in five people were interested in receiving advice about smoking cessation (16.9%, *n* = 199), and a third of the sample were interested in information about alcohol consumption (31.6%, *n* = 374).

**Table 2:** Interest in different types of lifestyle advice

	*n*	*%*	*Dichotomised %*
*Diet (n = 1187)*			
Very interested	319	26.8	
Somewhat interested	293	24.7	
A little interested	187	15.8	67.3
Not at all interested	275	23.1	
Not applicable	114	9.6	32.7
*Weight (*n *=* *1187)*			
Very interested	311	26.2	
Somewhat interested	275	23.2	
A little interested	196	16.5	65.9
Not at all interested	279	23.5	
Not applicable	126	10.6	34.1
*Physical activity (*n *=* *1181)*			
Very interested	281	23.8	
Somewhat interested	263	22.3	
A little interested	183	15.5	61.5
Not at all interested	329	27.9	
Not applicable	126	10.6	38.5
*Smoking (*n *=* *1177)*			
Very interested	74	6.3	
Somewhat interested	73	6.2	
A little interested	52	4.4	16.9
Not at all interested	337	28.7	
Not applicable	641	54.5	83.1
*Alcohol (*n *=* *1182)*			
Very interested	105	8.9	
Somewhat interested	142	12	
A little interested	126	10.7	31.6
Not at all interested	416	35.2	
Not applicable	392	33.2	68.4

### Current behaviour and interest in lifestyle advice at cancer screening

**Fig. 1:**
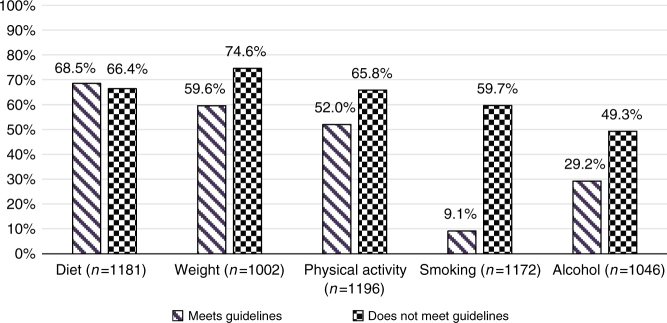
Proportion of participants willing to receive each type of lifestyle advice, by adherence to behavioural guidelines

Participants whose health behaviour fell short of recommendations were more likely to be interested in advice about weight, physical activity, smoking and alcohol consumption (Table 3; Fig. 1). Three quarters of participants in the overweight category expressed an interest in advice about keeping a healthy weight (74.6%) compared with 59.6% of participants classified as not overweight (OR 2.53, 95% CI 1.88–3.42, *p* < 0.001) (Fig. 1). Interest in advice about physical activity was expressed by 65.8% of people not taking part in 30 minutes of moderate activity five times per week, compared with 52.0% of people who were already physically active (OR 1.54, 95% CI 1.17–2.02, *p* = 0.002). Smokers had greater odds of interest in smoking cessation advice (59.7%) compared with non-smokers (9.1%; OR 16.23, 95% CI 10.70–24.62, *p* < 0.001). Participants who exceeded 14 alcoholic units per week were more likely to report interest in advice about alcohol consumption (49.3%) when compared with participants meeting alcohol consumption guidelines (29.2%; OR 2.35, 95% CI 1.56–3.53, *p* < 0.001). Analyses were re-run excluding those participants who felt the advice would not be applicable to them, and results were broadly unchanged. However, for interest in PA and alcohol, meeting the guidelines was not as strongly associated, and for interest in dietary advice, those who perceived themselves to be at lower risk were more likely to be interested in advice.

**Table 3:** Sociodemographic, psychological and behavioural predictors of interest in advice about diet, weight, physical activity, smoking, and alcohol consumption at cancer screening (adjusted logistic regression models)

	Diet		Weight		Physical activity		Smoking		Alcohol	
	**(***n* = 1086)		**(***n* = 923)		**(***n* = 1081)		**(***n* = 1078)		**(***n* = 1064)	
	OR (95% CI)	*p*	OR (95% CI)	*p*	OR (95% CI)	*p*	OR (95% CI)	*p*	OR (95% CI)	*p*
*Age*										
	0.99 (0.98–1.00)	0.147	0.99 (0.98–1.00)	0.100	0.99 (0.98–1.00)	0.035	0.99 (0.98–1.01)	0.313	0.98 (0.97–1.00)	0.007
*Gender*										
Male	REF	–	REF	–	REF	–	REF	–	REF	–
Female	1.04 (0.70–1.55)	0.846	1.16 (0.75–1.80)	0.497	1.20 (0.98–1.77)	0.361	1.33 (0.73–2.41)	0.353	0.67 (0.43–1.04)	0.075
*Ethnicity*										
White	REF	–	REF	–	REF	–	REF	–	REF	–
Non White	2.10 (1.27–3.45)	0.004	1.41 (0.83–2.40)	0.205	2.34 (1.42–3.85)	0.001	2.04 (1.18–3.51)	0.010	0.99 (0.64–1.52)	0.949
*Education*										
Degree level or above	REF	–	REF	–	REF	–	REF	–	REF	–
Below degree level	1.07 (0.79–1.46)	0.656	0.93 (0.67–1.30)	0.676	0.90 (0.67–1.22)	0.500	1.26 (0.81–1.95)	0.307	0.90 (0.66–1.22)	0.488
*Current or future screening attender*										
Current	REF	–	REF	–	REF	–	REF	–	REF	–
Future	1.31 (0.86–2.01)	0.204	1.45 (0.92–2.29)	0.110	1.42 (0.94–2.15)	0.095	1.13 (0.65–1.98)	0.661	1.13 (0.75–1.71)	0.555
*Comparative cancer risk*										
Same	REF	–	REF	–	REF	–	REF	–	REF	–
Lower	1.48 (1.07–2.05)	0.018	1.14 (0.81–1.60)	0.461	1.24 (0.91–1.70)	0.181	0.76 (0.48–1.19)	0.231	1.06 (0.77–1.45)	0.743
Higher	1.37 (0.92–2.03)	0.118	1.02 (0.66–1.56)	0.937	1.01 (0.70–1.47)	0.939	0.90 (0.54–1.51)	0.701	1.23 (0.70–1.52)	0.890
*Meets guidelines for behaviour in question*										
Yes	REF	–	REF	–	REF	–	REF	–	REF	–
No	0.96 (0.72–1.27)	0.762	2.53 (1.88–3.42)	<0.001	1.54 (1.17–2.02)	0.002	16.23 (10.70–24.62)	<0.001	2.35 (1.56–3.53)	<0.001
*Number of cancer risk factors recognised*										
	1.09 (1.03–1.14)	0.001	1.11 (1.05–1.17)	<0.001	1.07 (1.02–1.12)	0.008	1.09 (1.01–1.17)	0.018	1.12 (1.06–1.18)	<0.001

### Sociodemographic determinants of interest in lifestyle advice at cancer screening

The odds of reporting interest in advice about alcohol decreased with increasing age (OR 0.98, 95% CI 0.97–1.00, *p* = 0.007). Non-White participants were more likely to be interested in dietary and physical activity advice. Interest in dietary advice was expressed by 80.5% of non-White participants, compared with 65.5% of White participants (OR 2.10, 95% CI 1.27–3.45, *p* = 0.004). Similarly, 78.9% of non-White participants expressed interest in advice about physical activity compared with 59.2% of White participants (OR 2.34, 95% CI 1.42–3.85, *p* = 0.001). There were no associations between gender or education and interest in any of the five topics of advice.

### Psychological determinants of interest in lifestyle advice at cancer screening

Cancer risk factor awareness was positively associated with interest in advice about most lifestyle topics. With each additional cancer risk factor recognised, the odds of being willing to receive advice about diet (OR 1.09, 95% CI 1.03–1.14, *p* = 0.001), weight (OR 1.11, 95% CI 1.05–1.17, *p* < 0.001), physical activity (OR 1.07, 95% CI 1.02–1.12, *p* = 0.008), and alcohol consumption (OR 1.12, 95% CI 1.06–1.18, *p* < 0.001) increased. We also explored whether knowledge of individual risk factors were associated with interest in information about their corresponding topics of advice. People who recognised low fruit and vegetable consumption as a risk factor for cancer were more likely to want dietary advice at cancer screening (OR 1.48, 95% CI 1.10–1.98, *p *= 0.009). Recognition of alcohol consumption as a risk factor for cancer was associated with interest in advice about alcohol consumption (OR 1.70, 95% CI 1.30–2.22, *p * 0.001). Recognition of overweight (OR 1.12, 95% CI 0.84–1.51, *p *= 0.440), low physical activity (OR 1.24, 95% CI 0.95–1.62, *p *= 0.118), and smoking (OR 1.18, 95% CI 0.70–2.03, *p *= 0.530) were not associated with interest in their respective topics of advice. Comparative cancer risk perceptions were not strongly associated with interest in any of the topics of lifestyle advice.

## Discussion

This cross-sectional population-based survey of English adults found that people intending to participate in cancer screening are willing to receive advice about a range of behavioural cancer risk factors during the screening process. For most topics of advice, people not meeting health behaviour guidelines were more likely to be interested. In addition, non-White ethnicity, younger age, and greater cancer risk factor recognition were associated with interest in some topics of advice.

Providing lifestyle advice at cancer screening could be an effective strategy to promote behaviour change. However, if unacceptable to some people, the provision of lifestyle advice at cancer screening could reduce screening uptake, or increase socioeconomic inequalities in screening participation (CS, CV, SGS, JW & RJB; under review). One concern about delivering lifestyle advice at cancer screening is that screening non-attenders will not be reached.^8^ This is a problem because people from more deprived areas, non-White populations, and people with poorer health behaviours may be less likely to attend cancer screening.^11,34,35^ It was therefore encouraging that within our sample non-White participants who intend to take part in cancer screening in the future were more likely to report interest in information about diet, physical activity and smoking cessation. This may suggest that non-White participants who *do* participate in cancer screening may be particularly open to offers of other cancer prevention services and advice. Additionally, non-White participants may see cancer screening as an opportunity to address information needs that are not currently being met elsewhere. Interest in advice about alcohol consumption reduced with increasing age. This should be considered when designing behavioural programmes for the screening context as English adults aged 65 and over drink more frequently younger age groups.^36^ There were no associations between gender, educational status and interest in lifestyle advice, however, further research is needed to confirm this within other samples.

Current health behaviour was associated with interest in advice about physical activity, weight, smoking and alcohol consumption at cancer screening. This suggests that a tailored approach to intervention design, which takes current behaviour into consideration, may be best in this setting. Tailored advice has been delivered within the context of colorectal cancer screening, and has been successful at increasing reported fruit and vegetable consumption but did not increase physical activity or reduce alcohol consumption.^37,38^ Tailoring may be particularly important for less prevalent behaviours, such as smoking and excessive alcohol consumption.

Previous research suggests that health care professionals may be reluctant to deliver advice about topics such as weight, fearing that this advice may cause distress to patients.^39^ Reassuringly, three quarters of overweight participants in our sample were willing to receive advice about weight as part of cancer screening services. This is supports the finding that advice about weight in a primary care setting is considered appropriate and helpful by the majority of patients, and that interest in diet and weight advice at breast screening is greater among overweight women.^18,40^

Cancer risk factor awareness and risk perceptions were explored as potential determinants of interest in advice at cancer screening. Risk perceptions were not strongly associated with interest in any of the topics of advice. However, a single item measure of comparative cancer risk was used.^26,27^ Further research is needed to understand whether other aspects of risk, such as affective or experiential risk perceptions are associated with interest in lifestyle advice at cancer screening.^41^ Risk factor awareness was positively associated with willingness to receive advice about diet, weight, physical activity, and alcohol consumption. Previous research has highlighted the importance of providing convincing evidence about the link between lifestyle and cancer risk within cancer screening settings.^24^ This suggests that interventions delivered during the cancer screening process may benefit from increasing awareness of cancer risk factors.

This research has limitations. As stated in previous research conducted with this sample, participants were not recruited from screening settings so questions relating to the delivery of lifestyle advice at cancer screening were hypothetical (CS, CV, SGS, JW & RJB; under review). The well-documented intention-behaviour gap means it is likely that a number of people intending to participate in cancer screening will not take up the offer when invited,^42^ and willingness to receive lifestyle advice may differ between people who intend to participate in screening, and people who actually participate. Interest in lifestyle advice is high across breast, bowel and cervical screening programmes (CS, CV, SGS, JW & RJB; under review). However, in this research participants were asked to rate their interest in lifestyle advice at cancer screening in general, not for each cancer screening programme individually. Therefore it is not known whether interest in different topics of advice may vary between screening modalities. A further limitation to this research is the dichotomisation of responses to items assessing interest in each of the topics of advice. This involved grouping participants who answered “not at all interested” and “not applicable”. We reanalysed the data excluding participants who responded “not applicable” and results were broadly unchanged.

Our sample appear to be more health conscious than would be expected in the general population. For example, around half of our sample had a BMI > 25, compared with 61% of English adults in the general population.^5^ The health behaviours of screening attenders have been found to differ from non-attenders, which may also be the case for the screening intenders within our sample.^35^ In addition, survey respondents may also be healthier than the general population.^43^ However, measures of health behaviour used in this study may also impact the findings of this research. All health behaviour measures were self-reported, which may be subject to a number of biases.^44^. For example, we used fruit and vegetable consumption as a proxy of diet, however the accuracy of self-reported dietary data has been questioned.^45^ These self-reported measures were then used to gauge whether people were meeting guidelines. To classify people as meeting guidelines for alcohol consumption we used a cut off of 14 units, however, it is recognised that any alcohol consumption can increase cancer risk.^46^ Willingness to receive advice about alcohol consumption may vary depending on the leniency of the recommendation used.

In conclusion, the majority of people who intend to participate in cancer screening would be willing to receive advice about common cancer risk factors at this time. Advice appears to be acceptable to people who do not meet recommendations for health behaviours, and to non-White participants. Increasing cancer risk factor awareness and tailoring advice to current behaviour may provide an important basis for the development of interventions within the cancer screening setting.

## Availability of data and materials

Data will be made available to other researchers after the acceptance for publication of the main findings of the ABACUS survey. Data requests will be assessed on a case-by-case basis. Users will be required to complete a data sharing agreement.
